# Clinics

**Published:** 1874-06

**Authors:** 


### Clinics.

***Case of Epilepsy.*** *Rush Medical College. Diseases of Brain and Nervous System.* Service of Dr. WALTER HAY. Reported by Henry L. Harrington.

Patient, a woman, thirty-five years of age. Single. Five years ago was thrown from a horse, striking upon her head. Remained unconscious for an hour; suffered from the injury but a few hours. This was in September. The following April commenced having the attacks, at first in the night. Preceding the fit has experienced a sensation as if something were rising from the stomach to the throat. Generally cries out when attacked, and falls down, losing consciousness, and becomes convulsed, the muscles of the face and neck being first affected. Has sometimes bitten her tongue during the attacks. At times has no convulsion at all, but simply loses consciousness for a few moments, the face becoming pale. The attacks have continued to the present time, being most frequent at night. Says her memory is failing, and that her sight has become weakened. Suffers from headache a great deal. Has taken the bromide of potassium more than a year, not regularly, but when she felt that the fit was coming. The severe attacks have been ameliorated by the medicine, but the slighter ones have not been affected. Knows of no cases of the disease in her family. On examination, the patient is not well nourished; is anæmic; the pupils are dilated, tongue flabby and indented; appetite variable; bowels regular. She was ordered the following: Sodii Bromidi, oz. ij. Aquæ, O. ij. Of which to take one tablespoonful three times daily.

*Remarks.*—The case is interesting from the fact that both forms of the attack are present in the same case, and also illustrates the fact that the attack which occurs without any convulsions is the most obstinate and uncontrollable. In this case the predisposing cause probably consisted in the condition of the patient, and the exciting cause was the injury. The prognosis is unfavorable as regards a complete cure, but we may hope to ameliorate the symptoms by rendering the attacks less frequent.

CASE 2. Left hemiplegia. Man, aged thirty-three years. He has been a fireman for eight years. Six months ago he became suddenly paralyzed, without losing consciousness. He has since regained, to a certain extent, the use of his leg, and can walk, but has to use a cane, and drags the left foot. The arm is still nearly useless. At first the face was also affected, being drawn to the left side. Neither sight nor hearing have been at all affected, nor has intellection. He has had electricity applied, with but little benefit. The patient is not well nourished; face pale; pupils are dilated; tongue normal; appetite poor; bowels regular; urinary organs healthy. The circulation is very torpid upon the left side. He was ordered the following: R. Strychniæ (granules) *aa* gr. $\frac{1}{20}$, three times daily. And also the use of electricity in the form of the interrupted current.

*Remarks.*—The case is one of left hemiplegia, the face, however, being paralyzed upon the right side. It is probably due to cerebral hemorrhage upon the right side in the region of the pons varolii and crura cerebri. Considering the age and constitution of the patient, the prognosis is favorable, though it will take some months to effect a cure.

CASE OF LITTLE GIRL AGED TWELVE YEARS. The mother says the child has trouble in speaking and reading. Upon having her repeat the alphabet, it is noticed that she has difficulty in pronouncing the vowels, but not so with the labials. Her general health is poor; she does not play, seems too weak. Her face is pale; pupils dilated; conjunctiva has a pearly lustre; tongue smooth and pale; hands cold; appetite poor, and she does not sleep well; her bowels are regular; pulse seventy-two per minute, small and irregular. She has a dry cough, and at times has pain in the epigastric region, if she walks fast or runs. She does not eat fat meat or butter; does not like them. She was ordered the following: Strychniæ, gr. j.; Olei Morrhuæ, Syrupi Zingiberis, *aa* oz. iv. Of which she should take one teaspoonful after meals and at bedtime. Also to have good nutritious diet, and gentle exercise in the open air.

*Remarks.*—There is no organic lesion present, but the condition is one of hysteria. There is no paralysis of the lips or tongue. The trouble in pronunciation is due to an irregular supply of nerve force to the muscles of the larynx. Prognosis is favorable.

---

### *Obituary.*

At a called meeting of the Zanesville Academy of Medicine, held Saturday, May 2, to take action in relation to the death of its late Fellow, Dr. Jno. G. F. Holston, Sr., which took place at Washington, D. C., May 1, 1874, the following resolutions were adopted:

That we, whose occupation has been to relieve human suffering, are reminded that the time must come when our places on earth shall be vacated; therefore,

*Resolved*, That in the death of Dr. Holston, the Zanesville Academy of Medicine loses one of its prominent members, the profession at large an eminent physician and surgeon of extensive professional and literary culture, ripe experience, and accurate judgment, and society a warm-hearted, genial and generous member, whose life has been mainly devoted to the good of his fellow-beings.

*Resolved*, That we attend the obsequies of our deceased Fellow in a body.

*Resolved*, That we deeply sympathize with the family and relatives of the deceased.

*Resolved*, That the Corresponding Secretary transmit a copy of these resolutions to the family, the city press, and the medical journals.

A. E. BELL, Secretary *pro tem.* C. C. HILDRETH, Chairman.

